# Supplementary material for: Association between oral microbiome and seven types of cancers in East Asian population: a two-sample Mendelian randomization analysis
Source: Front Mol Biosci. 2023 Nov 21;10:1327893. doi: 10.3389/fmolb.2023.1327893 (PMC10702768; doi:10.3389/fmolb.2023.1327893)
Supplement: Supplementary file 1 [file DataSheet1.ZIP › Supplementary Materials/MR plots of saliva/saliva═╝/Gastric cancer/pheno.3362_to_gastric cancer_forest.pdf]

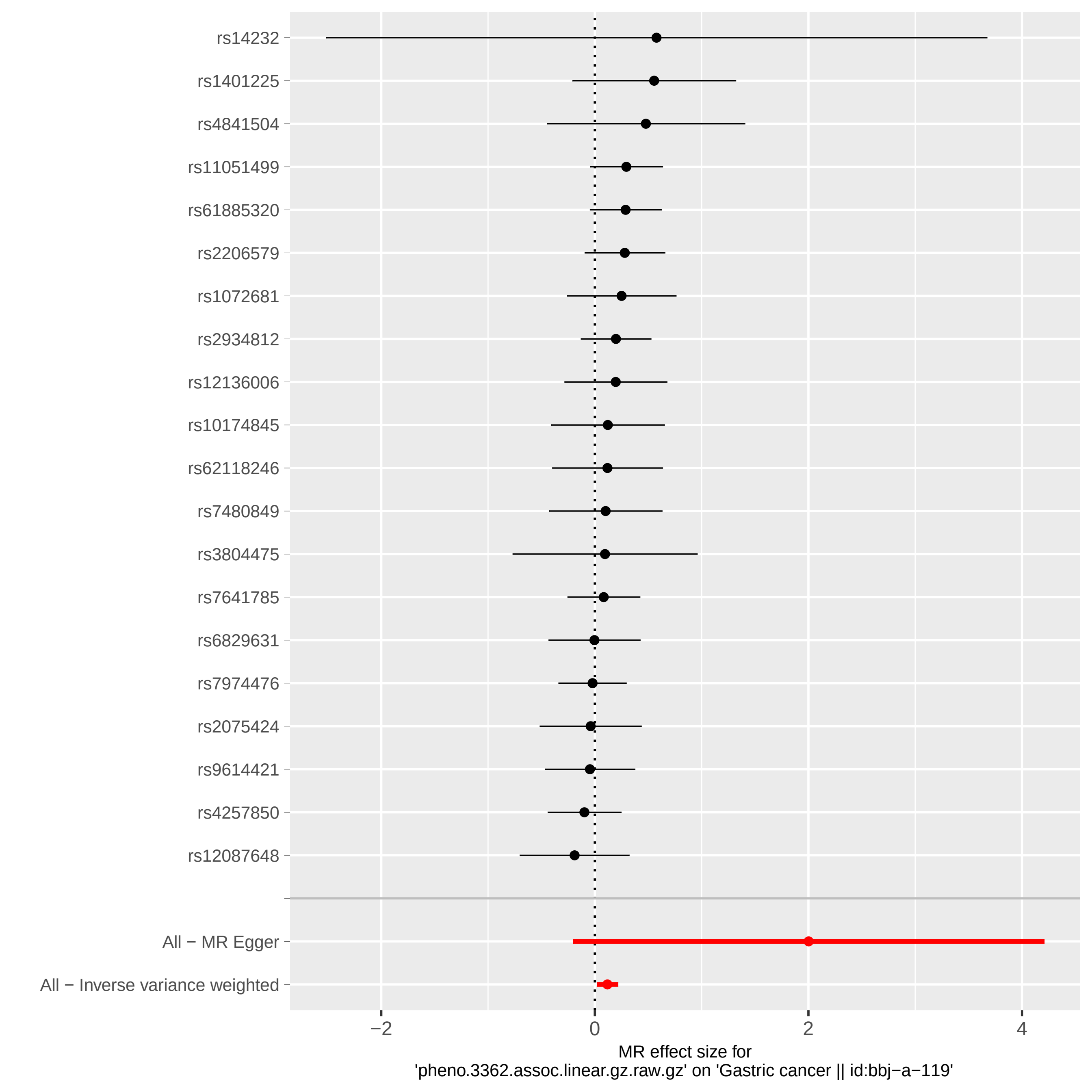

rs14232
rs1401225
rs4841504
rs11051499
rs61885320
rs2206579
rs1072681
rs2934812
rs12136006
rs10174845
rs62118246
rs7480849
rs3804475
rs7641785
rs6829631
rs7974476
rs2075424
rs9614421
rs4257850
rs12087648
All − MR Egger
All − Inverse variance weighted
−2
0
2
4
MR effect size for
'pheno.3362.assoc.linear.gz.raw.gz' on 'Gastric cancer || id:bbj−a−119'
